# Diagnostic Value and Application of Prenatal MRI and Ultrasound in Fetal Cleft Lip and Palate

**DOI:** 10.1155/2022/9410161

**Published:** 2022-05-18

**Authors:** Xin Yan, Guojing Xing, Xin Wang, Jun Li, Qiuhong Sun, Xiaojie Shang

**Affiliations:** ^1^Department of Ultrasound Medicine, Zibo Central Hospital, Zibo 255000, Shandong, China; ^2^Department of Imaging,The Fourth People's Hospital of Zibo, Zibo 255000, Shandong, China; ^3^Department of Neurosurgery, Zibo Central Hospital, Zibo 255000, Shandong, China; ^4^College of Medical Technology, Zibo Vocational Institute, Zibo 255300, Shandong, China

## Abstract

**Objective:**

The purpose was to explore the diagnostic value and application of prenatal magnetic resonance imaging (MRI) and ultrasound (US) in fetal cleft lip and palate.

**Methods:**

From January 2018 to December 2019, 39 pregnant women without normal fetal maxillofacial structure or with fetal maxillofacial deformity under US examination in our hospital were selected as the study subjects. Not knowing the clinical data of the pregnant women, MRI and US physicians performed diagnostic analysis on the MRI or US images of all the study subjects and analyzed the results of prenatal MRI and US diagnosis and postpartum follow-up to compare the diagnostic efficacy and confidence of MRI and US.

**Results:**

The follow-up found that there were 20 cases of cleft lip, 15 cases of cheilopalatognathus, 3 cases of cleft palate, and 1 case of unilateral cleft lip with alveolar cleft, with a total of 39 cases having cleft lip and palate deformity. MRI and US had the same efficacy in the diagnosis of cleft lip. As for cleft palates, the diagnostic accuracy of MRI (94.87%) was significantly better than that of US (48.72%, *P* < 0.001). The diagnostic confidence of fetal cleft lip and palate by MRI (89.73%) was significantly better than that of US (43.59%, *P* < 0.001). The AUC of US (0.597) was significantly less than that of MRI (0.940), indicating that the diagnostic accuracy of US was not as good as that of MRI (*P* < 0.05). The sensitivity and 1 − specificity of MRI were significantly higher than those of US.

**Conclusion:**

MRI is more accurate than US in the diagnosis of fetal cleft lip and palate, and MRI can be the preferred method for prenatal detection of cleft lip and palate, thus providing more accurate opinions and information for perinatal pregnant women.

## 1. Introduction

Cleft lip and palate refers to a disease that causes clefts of soft and bone tissues of the lip or palate of the fetus during embryonic development. Fetal congenital facial malformation is a kind of congenital body surface malformation [1–3]. The malformation can appear at the birth of fetuses, which is not conducive to the growth and development of the children. Fetal cleft lip and palate is a common congenital facial malformation, which not only affects the appearance of children but also causes malnutrition in children with the difficulty of sucking milk, having a certain impact on the psychology of parents and children [4]. At the same time, most scholars believe that diabetes and malnutrition in pregnant women during pregnancy are the main factors contributing to fetal cleft lip and palate, but previous reports have shown that genetic factors and viral infection are also the main factors causing this disease [5]. In recent years, the reported incidence of cleft lip and palate has increased in various countries, with the incidence of 1/495–1/1995 worldwide and 1.8% in China. Epidemiological studies in developed countries such as the United States and Europe have shown that the incidence is higher in the yellow race than in the black and white races, and the incidence is influenced by geographical, ethnic, and socioeconomic factors [6]. Ultrasound (US) is currently the preferred method for clinical diagnosis of fetal cleft lip and palate. However, its detection results have certain limitations due to factors such as gestational age, amniotic fluid volume, and maternal obesity of pregnant women. Several studies have pointed out that MRI has a positive role in prenatal diagnosis of fetal cleft lip and palate [7, 8]. In addition, with the characteristics of multiple imaging, no ionizing radiation, and relatively objective diagnosis, MRI is less affected by the clinical experience of the operators, and its visual field is less affected by the fetal position. Therefore, MRI is widely used in the examination of fetal malformation. The purpose of this study was to explore the diagnostic value and application of prenatal MRI and US examinations in fetal cleft lip and palate, aiming to objectively analyze the accuracy of MRI and US in the diagnosis of fetal cleft lip and palate, specifically reported as follows.

## 2. Materials and Methods

### 2.1. General Information

From January 2018 to December 2019, 39 pregnant women without normal fetal maxillofacial structure or with fetal maxillofacial deformity under US examination in our hospital were selected as the study subjects. This study was in line with the principle of the Declaration of Helsinki [9].

### 2.2. Inclusion Criteria and Exclusion Criteria

#### 2.2.1. Inclusion Criteria

1. All pregnant women voluntarily received prenatal MRI and US diagnosis on the same day.
2. All of them voluntarily accepted postpartum neonatal maxillofacial examination or the test results.
3. This study was approved by the hospital ethics committee and the pregnant women signed a consent form after being informed.
4. Pregnant women had a family history of cleft lip and palate.

#### 2.2.2. Exclusion Criteria

1. Pregnant women with severe pregnancy complications.
2. Pregnant women with two or multiple pregnancies.
3. Pregnant women who were transferred to other hospitals midway and no postdelivery results were obtained.

### 2.3. Methods

#### 2.3.1. Diagnostic Confidence Scale and Cleft Lip and Palate Classification

The diagnostic results of cleft palate were classified into five grades, including definitely cleft palate (grade 5), probably cleft palate (grade 4), uncertain (grade 3), probably not cleft palate (grade 2), and certainly not cleft palate (grade 1), scored as 5, 4, 3, 2, and 1, respectively. Negative diagnosis was not cleft palate, and the nodes of diagnosis were definitely cleft palate, probably cleft palate, uncertain, and probably not cleft palate. Definite diagnosis included certainly not cleft palate and definitely cleft palate, with 100% of diagnostic confidence. Uncertain diagnosis included probably cleft palate, uncertain, and probably not cleft palate, with diagnosis confidence as 75%, 50%, and 25%, respectively.

In this study, MRI and US images were independently diagnosed by two diagnostic physicians with more than 10 years of diagnostic experience in our hospital. The diagnostic result was selected if two diagnostic physicians had same diagnosis. When the results were different, another two diagnostic physicians took a result after discussion. None of the four diagnostic physicians knew the clinical data and indicators of pregnant women and fetuses before diagnosis.

According to the different fetal development and cleft palate locations, cleft lip and palate was divided into cleft lip (CL), cleft palate (CP), cleft lip with alveolar cleft (CLA), and cleft lip with complete cleft palate (CLP), bilateral and unilateral.

#### 2.3.2. US Detection Method

Experienced US diagnostic physicians used standardized methods to obtain the two-dimensional images of the fetal abdomen, chest, and limbs and to obtain the two-dimensional and three-dimensional images of the fetal head. Then, the images were stored in the picture archiving and communication system (PACS). The color Doppler ultrasonic diagnostic system (model: Philips HD7) was used for ultrasound examination with the activation of fetal protection key. The two-dimensional ultrasound emission capacity energy was less than 100 mW/cm^2^, with 20 Hz–40 Hz as the frequency of exploration.

#### 2.3.3. MRI Detection Method

The Amira 1.5 T superconducting scanner (Siemens) was used for MRI detection with spine coil and body coil. True FLSP (true fast imaging with steady-state precession) was used, with the scan reference data set as 1.93 ms of TE, 612 ms of TR, 256 × 256 of rectangle, 79° of flip angle, 4.0 mm of layer thickness, 1.00 of SNR, −50% of scanning interval, 380 mm of FOV, Averages 1, 484 Hz/PX of bandwidth, and 19 s of scan time. Pregnant women breathed freely in the supine position. The scan was performed in sagittal, axial, and coronal directions. Median sagittal/coronal scanning included maxillofacial region and brain. The axial scan was parallel to the deciduous dentition and included the fetal throat to the lower margin of mandible. The captured images were uploaded to picture archiving and communication system (PACS).

### 2.4. Observation Indexes

The postpartum follow-up results, the diagnostic accuracy of MRI and US, and diagnostic classification results were observed and recorded.

### 2.5. Statistical Processing

The data in this study were processed and analyzed by the data software SPSS20.0. The area under the curve (AUC) and receiver operating characteristic (ROC) were used to compare the diagnostic efficacy of prenatal MRI and US in the diagnosis of cleft lip and palate. The measurement data were measured by the *t*-test, expressed by ($\bar{x}$ ± *s*), and the count data were tested by *X*2, expressed by (*n* (%)). The difference was statistically significant when *P* < 0.05.

## 3. Results

### 3.1. Comparison of General Data

The baseline data of all subjects are shown in Table 1.

### 3.2. Postpartum Follow-Up Results

The follow-up found that 39 fetuses suffered from cleft lip and palate deformity, in which parents of 36 cases were informed of the detection results and parents of 3 cases were informed of autopsy results. There were 20 cases of cleft lip (18 unilateral and 2 bilateral), 15 cases of cheilopalatognathus (14 unilateral and 1 bilateral), 3 cases of cleft palate, and 1 case of unilateral cleft lip with alveolar cleft. None of the 39 children had concurrent malformations.

### 3.3. Diagnostic Accuracy of MRI and US

MRI and US had the same efficacy in the diagnosis of cleft lip. As for cleft palates, the diagnostic accuracy of MRI (94.87%) was significantly better than that of US (48.72%, *P* < 0.001), as shown in Table 2.

### 3.4. Follow-Up and Diagnostic Grading Results of MRI and US

In MRI, there were 35 cases of definite diagnosis, 1 case of missed diagnosis, and 3 cases of uncertain diagnosis (accounting for 7.69%). In US, there were 17 cases of definite diagnosis, 2 cases of missed diagnosis, and 20 cases of uncertain diagnosis (accounting for 51.28%). It could be seen that the diagnostic confidence of fetal cleft lip and palate by MRI (89.73%) was significantly better than that of US (43.59%), with statistical significance (*P* < 0.001). The follow-up and diagnostic grading results of MRI and US are shown in Table 3.

### 3.5. Analysis of AUC and ROC of MRI and US

**Figure 1:**
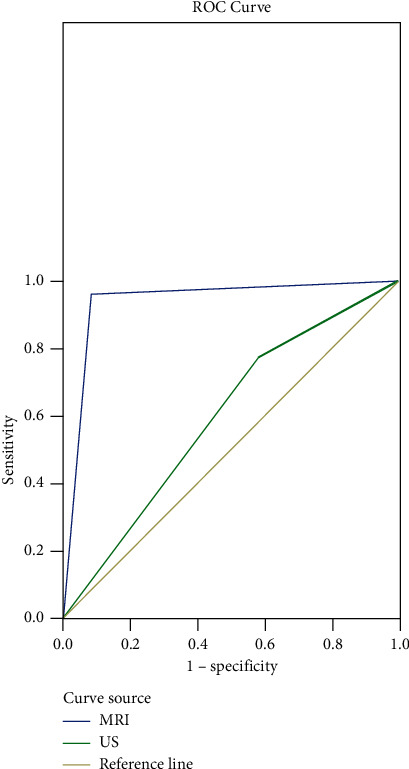
ROC analysis of MRI and US in the diagnosis of fetal cleft lip and palate.

The AUC of US (0.597) was significantly less than that of MRI (0.940), indicating that the diagnostic accuracy of US was not as good as that of MRI (*P* < 0.05), as shown in Table 4 and Figure 1.

### 3.6. Comparison of Sensitivity and 1 − Specificity

The sensitivity and 1 − specificity of MRI were significantly higher than those of US, as presented in Table 5.

## 4. Discussion

One of the most common facial congenital malformations is cleft lip and palate [10–12]. Relevant studies indicate that more than 50% of children with cleft lip suffer from cleft palates, but their causes are different [13]. During the fetal development, there are 2 mandibular processes, 2 maxillary processes, and 1 nasopalatine process around the original mouth of the embryo, and several protrusions will grow out along with the development. After 7 weeks of development, if the two globular processes from the nasopalatine process are not fused, a cleft lip will arise in the middle of the upper lip. If the globular process does not fuse with the maxillary process on one side, a unilateral cleft lip will arise. If the globular process does not fuse with the maxillary processes on both sides, a bilateral cleft lip will arise [14]. The palates are divided into secondary palate and primary palate. The primary palate is the anterior part of the palate, which is a small part of the palate and formed by fusion of the anterior palatal processes on both sides of the globular process. The secondary palate is formed by the fusion of two palatal processes toward the midline, which also fuses with the primary palate. After 8 weeks of development, if the nasal septum and bilateral palatal processes of the secondary palate do not fuse with the primary palate with bilateral anterior palatal processes, cleft palate will occur. Primary cleft palate is clinically referred to as alveolar cleft [15].

US is the preferred screening method for prenatal maxillofacial deformity clinically. However, it is difficult to make a clear judgment for US detection in cases of excessive obesity of pregnant women and small amniotic fluid volume. In addition, it is difficult for US detection to display clear images because echoes from protrusion of fetal maxillary alveolar and other parts are easily occluded. Relevant reports indicate that it is very safe to perform MRI detection on fetuses over 3 months of gestation, which can display the fine structure of the fetuses in any direction with a high anatomical resolution. Therefore, MRI is mostly used in clinic for further detection when US cannot be used for detection [16].

In clinical diagnosis, MRI is characterized by large soft tissue resolution, non-ionizing radiation, and large visual field imaging without limitations of maternal obesity, small amniotic fluid volume, and other factors. The rapid imaging of MRI greatly reduces fetal motion artifacts. According to the study of Werner et al. [17], MRI has a positive effect on prenatal imaging detection. Although prenatal MRI detection is widely used in clinic, MRI is not included in routine prenatal screening at present. MRI diagnosis is often performed after the US detection results in the diagnosis of cleft lip and palate are obtained, which directly improves the diagnostic efficiency of MRI. In order to analyze the diagnostic value and application of prenatal MRI and US examinations in fetal cleft lip and palate, 39 pregnant women without normal fetal maxillofacial structure or with fetal maxillofacial deformity under US examination in our hospital from January 2018 to December 2019 were selected as the study subjects. MRI and US physicians performed diagnostic analysis on the MRI or US images of all the study subjects without knowing the clinical data of the pregnant women, so that the diagnostic results of cleft lip and palate were more objective.

39 cases were studied in this study, with a gestation of 20–38 weeks. The results of this study found that MRI and US had the same diagnostic efficacy with the diagnostic accuracy, specificity, and sensitivity all as 100%. As for cleft palates, the diagnostic accuracy of MRI (94.87%) was significantly better than that of US (48.72%), with statistical significance (*P* < 0.001). According to the “The prenatal diagnosis and classification of cleft palate: the role and value of magnetic resonance imaging” studied by Zheng et al. [18], the diagnostic rates of US and MRI were 59.09% and 92.05%, respectively, which were consistent with the conclusion of this study, thus indicating that MRI diagnosis was more accurate than US diagnosis.

In this study, both MRI and US could accurately diagnose unilateral cleft lip with complete cleft palate. MRI could clearly show the maxillofacial structure and brain parenchyma of a fetus with maternal obesity and isolated cleft palates without holoprosencephaly at 25 weeks of gestation, while US could not. There were 6 cases of missed diagnosis and 14 cases of uncertain diagnosis in US, mainly because maternal obesity covered the maxillofacial bones. There were 2 cases of uncertain diagnosis in MRI mainly due to the unclear display of the lip and palate caused by the artifacts generated by fetal motion. MRI diagnosis in this study showed that the cleft lip and palate of fetuses over 20 weeks of gestation was not significantly correlated with the gestational age of pregnant women while artifacts of fetal movement were the main influencing factors of MRI detection. The main factor affecting US detection was whether the maxillofacial area was covered or whether the mother was obese. In addition, the ROC curve analysis showed that the curve area of MRI was larger than that of US, suggesting that MRI can provide more information, with a lower number of misdiagnosis and missed diagnosis. Therefore, MRI diagnosis can be added to provide more accurate opinions for pregnant women when the results of US are unclear. This study also has some inadequacies. First, due to the limitations of relevant conditions, this study has a small sample size and limited sample source and lacks representativeness. Second, the different skills of staff in MRI and US examinations have a certain impact on the research results. Finally, this study is based on the patients within the region and did not include a sufficient number of patients from other provinces, so the results may be affected by the small sample size and regional culture.

In conclusion, MRI is more accurate than US in the diagnosis of fetal cleft lip and palate, and MRI can be the preferred method for prenatal detection of cleft lip and palate, thus providing more accurate opinions and information for perinatal pregnant women.

## Figures and Tables

**Table 1:** Statistics of baseline data of all subjects (*n* (%)).

Items	*N*	Percentage
Age		
21–29 years old	31	79.49
30–40 years old	8	20.51
Average gestational age (weeks)	29.42 ± 5.56	—

Occupation		
Teachers	10	25.64
Financial practitioners	11	28.21
Accountants	12	30.77
Individual households	4	10.26
Other	2	5.13

Family income		
≥3000 yuan/(month·person)	30	76.92
<3000 yuan/(month·person)	9	23.08

Residence		
Urban area	25	64.10
Rural area	14	35.90

Education		
University	25	64.10
Middle school	12	30.77
Primary school	2	5.13

Nations		
Han	35	89.74
Other	4	10.26

**Table 2:** Diagnostic accuracy of MRI and US (*n* (%)).

Diagnosis	Misdiagnosis	Missed diagnosis	Uncertain diagnosis	Diagnostic accuracy
MRI diagnosis	0	0	2	(37/39) 94.87%
US diagnosis	0	6	14	(19/39) 48.72%
*X* ^2^				20.51
*P*				<0.001

**Table 3:** Follow-up and diagnostic grading results of MRI and US.

Number of cases	MRI diagnostic confidence grading	US diagnostic confidence grading	Follow-up results
12	1	1	11 cases of CL, 1 case of CLP
1	4	1	1 case of CLP
3	5	1	1 case of CP, 2 cases of CLP
7	1	4	7 cases of CL
2	3	3	2 cases of CP
4	5	3	1 case of CL, 1 case of CLA and 2 cases of CLP
1	1	4	1 case of CL
1	4	4	1 case of CLP
7	5	4	7 cases of CLP
1	5	5	1 case of CLP

**Table 4:** Statistical results of fetal cleft lip and palate diagnosed by MRI and US.

Progressive 95% confidence interval					
Detection variables	Area	Standard error^a^	Progressive sig.^b^	Upper limit	Lower limit
MRI	0.940	0.051	<0.001	0.000	1.000
US	0.597	0.102	0.338	0.397	0.797

a = under nonparametric hypothesis; b = null hypothesis, real area = 0.5.

**Table 5:** Comparison of sensitivity and 1 − specificity.

Detection variables	Positive^a^ if greater than or equal to	Sensitivity	1 − specificity
MRI	−1.0000	1.000	1.0001
	0.5000	0.963	0.083
	2.0000	<0.001	<0.00

US	−1.0000	1.000	1.000
	0.5000	0.778	0.583
	2.0000	<0.001	<0.001

a = under nonparametric hypothesis.

## Data Availability

The data used to support the findings of this study are available from the corresponding author upon request.
